# Application of peripherally inserted central catheters in critically ill newborns experience from a neonatal intensive care unit

**DOI:** 10.1097/MD.0000000000015837

**Published:** 2019-08-09

**Authors:** Renfeng Li, Xia Cao, Tian Shi, Lei Xiong

**Affiliations:** Neonatology Department, Chongqing Health Center for Women and Children, Chongqing, China.

**Keywords:** catheterization, catheter-related infections, catheters, indwelling, infant, newborn, peripheral

## Abstract

Peripherally inserted central catheters (PICCs) can provide nutritional and medical support for very low birth weight or critically ill newborns. The aim of this study was to retrospectively analyze the use of PICCs in our clinic for critically ill newborns to evaluate the relationship between catheter related factors and the occurrence of complications.

Retrospective analysis was conducted for all newborns consecutively admitted at the Neonatal Intensive Care Unit (NICU), Chongqing Health Center for Women and Children, who underwent PICC insertion between May 2011 and March 2018. Data collected included total puncture success rate, one puncture success rate, infection rate, complication rate, unplanned catheter withdrawal rate, device days, and catheter indwelling time.

Five-hundred eighty-eight infants (304 males and 284 females) aged 3.4 ± 3.9 days, mean gestational age of 30.9 ± 2.7 weeks and a mean body mass of 1.38 ± 0.47 kg at insertion were included. Total puncture success rate was 99.65%, one puncture success rate was 77.77%. The mean catheter retention was 13.6 ± 6.7 days: more than 30 days in 15 (2.61%) cases, 20 to 30 days in 60 (10.43%) cases, 10 to 19 days in 372 (64.70%) cases, and 62 days in 1 case. Complications occurred in 63 (10.71%) cases: with PICC insertion within 24 hours after birth in 29 (15.43%), within 48 hours in 13 (6.63%), and after 48 hours in 21 (10.99%) cases. Catheter tip culture was positive in 3 cases and there was 1 case of catheter-related bloodstream infection.

Nursing measures of the maintenance of body temperature and the evaluation of blood vessels were important conditions for improving the success rate of one puncture in critically ill neonates. PICC catheterization as early as 48 hours will not increase the difficulty of PICC puncture. Nor did it increase the incidence of PICC complications.

## 1 Introduction

A peripherally inserted central catheter (PICC) refers to a catheter that enters the body at a peripheral site with its tip fixated at the superior vena cava. PICC is used for medium- to long-term intravenous infusion therapy or infusion therapy with stimulant drugs.^[1]^ When newborns have very-low birth weight or are critically ill they often cannot obtain enough nutrition from the gastrointestinal route within the weeks after birth and usually require infusions of hyperosmotic or irritant drugs.^[2]^ Repeated intravenous catheterization increases the pain and the chance of infection.^[3]^ Thus, establishment of long-term venous access with hyperosmotic resistance is crucial for rescue treatment in these infants.

PICC insertion is a safe, convenient and effective technique characterized by its simplicity, high success rate, with few complications, and resistance to hyperosmolarity.^[4,5]^ Therefore, the use of PICC can reduce excessive stimuli to the infants and ensure the supply of intravenous nutrition and timely administration of medication during rescue. However, the long-term retention of a PICC makes incidence of catheter-related bloodstream infection (CRBSI) highly likely^[6]^ and it remains unclear how long PICCs can remain indwelling, as they were first intended for short term vascular access.^[7]^ One study suggests that they should be replaced if required after 35 days,^[7]^ while another suggests that uninfected PICCs should not be replaced.^[8]^ Another risk factor for complications during the use of PICCs is the insertion site^[9,10]^ But the risks of complications may be higher, and the result of the complications may be more severe in critically ill infants than in those with low-birth weight.^[11]^

In our clinic, we treat many critically ill newborns and they often receive PICC. Therefore, we retrospectively analyzed the data from our clinic to establish the occurrence of complications and catheter-related risk factors with the use of PICC in critically ill newborns.

## 2 Patients and methods

A retrospective analysis was conducted for all the newborns who were consecutively admitted at the Neonatal Intensive Care Unit (NICU), the Neonatal Department, Chongqing Health Center for Women and Children, and underwent PICC insertion between May 2011 and March 2018. The inclusion criteria were: Infants who accepted PICC insertion for the first time and had complete catheterization records. The exclusion criteria were: Patients who had severe infections (such as sepsis, or necrotizing enterocolitis) prior to PICC insertion, who had the PICC withdrawn within 48 hours after the insertion, who died from illness during hospitalization, who signed for discharge or transfer to another hospital, and who had incomplete records.

This study was approved by the institutional review board of the Chongqing Health Center for Women and Children.

### 2.1 PICC procedure

After obtaining the signed informed consents from the patients, nurses qualified for PICC insertion performed the insertion using the 1.9Fr PICC catheter (BD, USA). A preoperative routine coagulation test was performed. The preferred site of puncture was the right basilic vein, followed by middle elbow vein, axillary vein and cephalic vein. Prior to the puncture, distance between the pre-marked site of puncture and the right sternoclavicular joint along the venous passage was measured as the catheter length. After placing the infants onto the preheated far-infrared radiation table for appropriate warmth, iodophor skin disinfectant (Jianzhisu, Beijing, China) was used to disinfect the whole torso, shoulder and chest at the puncture side while sterile towel drape was placed to maximize the sterile barrier. Postoperative X-ray examination was performed to position the arm of the infant so that the tip of the PICC catheter was at the deepest site and the position of the tip could be identified.

### 2.2 PICC care and dressing changes

All the catheter-related information, including catheter name, catheter type, name of punctured vein, failed/successful puncture, operative time, insertion depth, length of exposed catheter, bilateral arm circumference, and condition of the puncture site, were recorded postoperatively. Dressings were changed once every week or whenever there was any curling, damage or contamination to the dressing. Dressing changes were completed by 2 nurses simultaneously to avoid catheter prolapse or migration. The date of the change was recorded on the dressing. The heparin cap was changed weekly, and attention was paid to the position of the catheter to avoid internal migration. Aseptic operation was followed strictly, the infants were cared for by specially designated nurses, and the catheter was disinfected with iodophors twice when changing the heparin cap and reconnected after drying. Fluid preparations were performed under strict aseptic operation. The dressing was changed after the first 24 hours following the puncture and nurses observed and recorded whether or not there was bleeding and/or signs of infection at the puncture site. If bleeding was identified, the dressing was changed immediately, and a sterile gauze was used for covering and hemostasis. The gauze applicator was changed every 48 hours. Changes in the patient's basic conditions such as body temperature, left and right arm circumference, length of the exposed catheter, and puncture site, as well as changes during the subsequent catheterization, were recorded.

### 2.3 Data collection

Monitoring indicators included: total puncture success rate, one puncture success rate, incidence rate of infection, incidence rate of complications (catheter obstruction, mechanical phlebitis, catheter prolapse, and venous thrombosis), rate of unplanned catheter withdrawal, device days, and catheter indwelling time. Unplanned catheter withdrawal refers to premature catheter withdrawal due to any reason, such as catheter obstruction, leak, detachment or prolapse. Catheter-related bloodstream infection (CRBSI) was defined as the presence of bacteremia or fungalemia, accompanied by infection manifestations such as fever (>38°C), chills or hypotension, in patients with intravascular catheter or within 48 hours after catheter withdrawal; while no definite origins of infections or venous thrombosis can be identified apart from the intravascular catheter^[12]^

### 2.4 Statistical analysis

The SPSS 10.0 software (SPSS Inc., Chicago, IL) was used for statistical analysis. Measurement data was presented as mean ± standard deviation (SD) were analyzed by *t* test while count data were presented as median and range and analyzed by the *χ*^2^ test, with a 2-sided *P* < .05 indicating a statistically significant difference.

## 3 Results

### 3.1 Baseline data

A total of 588 infants (304 males and 284 females) with a mean gestational age of 30.9 ± 2.7 (range: 24–41^+4^ weeks) weeks and a mean body mass of 1.38 ± 0.47 kg (range at birth: 0.49–5.07 kg) at insertion were included. The patients’ mean age at insertion was 3.4 ± 3.9 days (range: 3 hours to 48 days) and mean body mass at insertion was 1.38 ± 0.47 kg (range: 0.49–5.07 kg). Of the 588 cases, the total puncture success rate was 99.65% and the one puncture success rate was 77.77%. Of the 131 successful cases with more than one puncture, 85 (64.89%) required 2 punctures and 21 required 3 punctures. See Table 1 for full details.

**Table 1:**
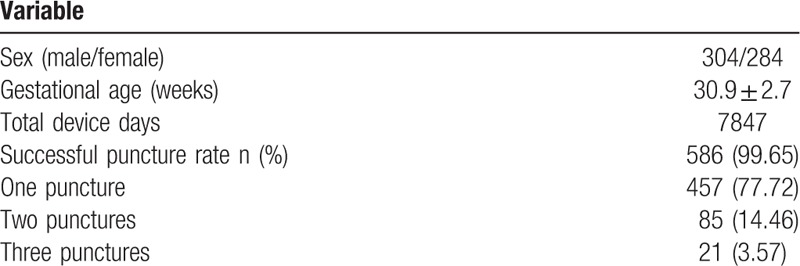
General patient data.

### 3.2 PICC time

The total number of days the PICC device was used was 7847 days, with a mean catheter retention duration of 13.6 ± 6.7 days (range: 1–62 days). The catheter was retained for more than 30 days in 15 (2.61%) cases, 20 to 30 days in 60 (10.43%) cases, 10 to 19 days in 372 (64.70%) cases, and 62 days in 1 case. Insertion was successful in 190 cases within 24 hours after birth, 198 cases within 24 to 48 hours after birth, and 200 cases after 48 hours after birth (Table 2).

**Table 2:**
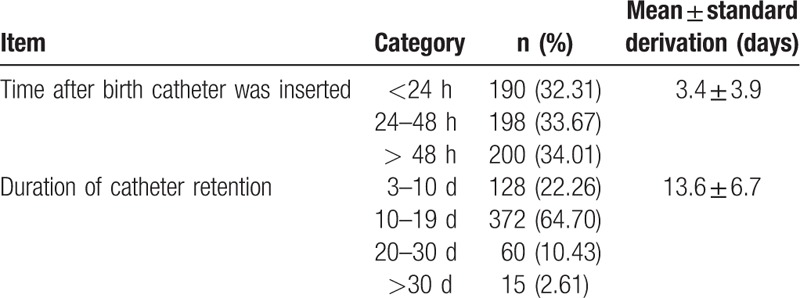
Catheter data.

### 3.3 Unplanned catheter withdrawal

Cases of unplanned catheter withdrawal (n = 28) accounted for 4.87% of all the cases. Reasons for withdrawal included unexpected discharged signed by the parents (n = 14), failed punctures (n = 2) and suspicious infections (n = 1).

### 3.4 Complications

Complications occurred in 63 (10.71%) cases, including 12 (2.04%) cases of catheter obstruction, 25 (4.25%) cases of mechanical phlebitis, 14 (2.38%) cases of catheter prolapse, 2 (0.34%) cases of venous thrombosis, 3 (0.51%) cases of catheter malposition, and 5 (0.85%) cases of pleural effusions. Complications occurred in 29 (15.43%) cases with PICC insertion within 24 hours after birth and 21 (10.99%) cases with insertion after 48 hours after birth and no significant difference was found between the 2 (*P* = .203). Thirteen cases (6.63%) with insertion within 24 to 48 hours after birth suffered from complications. Catheter tip culture was performed in 576 cases, with 3 positive cases and 1 case of CRBSI (Table 3). Blood culture was negative in all the PICC infants. Of the 3 cases with positive results from the catheter tip culture, Staphylococcus was identified, while blood analysis results and C-reaction protein levels were normal, blood culture was negative, and no symptoms of infection such as fever or chills were present in the patients during their hospitalization; therefore, catheter bacterial colonization was suspected.

**Table 3:**
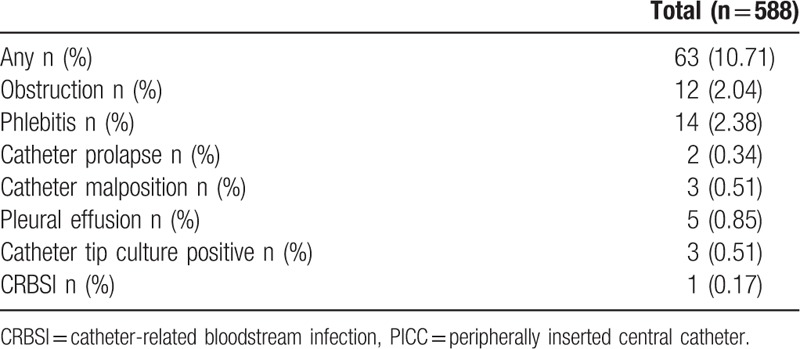
Complications during PICC.

## 4 Discussion

The aim of this study was to present a retrospective analysis of critically ill newborns who received PICC. We wanted to discover if there was a relationship between the catheterization time and complications in these patients. The results show that there was a total complication rate of 10.71% in this population that had a mean indwelling of the catheter of 3.6 ± 6.7 days. The catheter was in place for more than 30 days in 15 (2.61%) cases, 20 to 30 days in 60 (10.43%) cases, 10 to 19 days in 372 (64.70%) cases, and 62 days in 1 case. Complications occurred in 63 (10.71%) cases: with PICC insertion within 24 hours after birth in 29 (15.43%), within 48 hours in 13 (6.63%), and after 48 hours in 21 (10.99%) cases. There was only 1 case of CRBSI. This data suggests that earlier PICC (within 24 hours after birth) use did not significantly increase the rate of complications in this population.

PICC insertion is a safe, convenient, and effective technique. Based on previous experience, PICC insertion should be performed in newborns 2 to 3 days after their birth when the disease status is steady. For very-low/ultra-low birth weight infants after birth, the umbilical vein is usually selected for insertion to rescue and treat the infants; however, preterm infants are susceptible to functional intestinal obstruction, intestinal dilation, and increased intestinal pressure due to gastrointestinal immaturity, poor gastrointestinal motility and low local immune response. Meanwhile, a preterm infant's poor function in blood pressure regulation and portal hypertension induced by malposition of the umbilical vein catheter will obstruct the gastrointestinal blood return and cause gastrointestinal congestion, making umbilical catheterization another high-risk factor for necrotizing enterocolitis.^[13]^ Thus, early PICC insertion is critical for reducing postnatal complications and increasing survival rates of very-low birth weight infants, and it is also an extremely important approach to rescue of infants. In this study, 13 (11.11%) of 117 cases with PICC inserted within 24 hours had complications, 16 (15.53%) of 103 cases with PICC inserted within 48 hours had complications, and no statistical difference was found between the two groups (*P* > .05). We believe that our results may be affected by the small sample size and therefore we will continue the study. Nevertheless, earlier application of PICC insertion is another effective means to rescue infants and relieve their pain.

While PICC is critical to the rescue treatment of infants, repeated punctures increase the chance of infection and pain and cause pain-induced severe complications such as intracranial bleeding especially in very-low birth weight infants;^[14,15]^ therefore, it is extremely important to increase the puncture success rate. In the present study, the overall success puncture rate was 100% and the one puncture success rate was 81.19%. Since all the PICC insertions were completed by the same nurse, lack of dexterity can be ruled out. We believe that placing the infants onto a preheated radiation table contributes to vasodilation and venipuncture. Accurate position of the catheter tip is crucial to the safety and treatment of the infants and also key to the improvement of one puncture success rate. However, there is no direct position available during the insertion process and catheter length is estimated by the measurement of the body surface projection of the infant in clinical practice. The conventional measurement method is to measure the distance between the pre-marked site of puncture and the right sternoclavicular joint and the length extending downwards to the 3rd intercostal space. Other studies also reported a different method, namely the “linear external measurement”, which measures the distance between the pre-marked site of puncture and ipsilateral sternoclavicular joint and then measures the horizontal distance to the lateral edge of the contralateral sternal end.^[16]^ In this study, we found that the length estimated by the conventional method or the linear external measurement was usually longer than the actual length of the vascular passage. We therefore selected the distance between the pre-marked site and the right sternoclavicular joint along the venous passage, which helped reduce the chance of inserting too the catheter too deeply and increasing the first insertion success rate.

PICC insertion is mainly indicated for preterm infants, very-low/ultra-low birth weight infants or critically ill infants. Those patients are susceptible to nosocomial infections due to the immature function of the tissues and organs and low immune function; while the long retention of a PICC also makes incidence of CRBSI highly likely. CRBSI is the most severe complication following PICC puncture. It was reported that the incidence of CRBSIs increased by 14% per day during the first 18 days after PICC insertion, reversed from days 19 through 35, and increased again by 33% per day after 36 days of insertion; therefore, it is suggested to carefully evaluate whether or not the continuation of PICC insertion is necessary on day 35.^[7]^ In this study, the longest retention time was 62 days and 1 case had CRBSI. Of the 335 cases who had investigation of catheter tip culture, 3 (9%) cases were positive. For the infants with positive culture results, their blood analysis results and C-reaction protein levels were normal, blood culture was negative, and no symptoms of infection such as fever or chills were present during their hospitalization; therefore, catheter bacterial colonization was suspected. It was found recently that, although use of antibiotic-based catheter lock solutions reduces the incidence of CRBSI it can result in adverse events such as fungal infection, increased drug resistance or drug allergy.^[17,18]^ In our case, we used 6.25 U/ml heparin sodium for catheter locking and found no infection. The *Guide for prevention and control of catheter-related bloodstream infection 2011 version*^[19]^ suggests that although aseptic operations should be maximally achieved, for patients with history of CRBSI requiring long-term catheter insertion, antibiotic-based catheter lock solutions (type II) can be used. We do not recommend routine use of antibiotic-based catheter lock solutions in PICC insertion at our neonatal intensive care unit.

In the present study, 1 case experienced catheter withdrawal due to suspicions of CRBSI but the sequential catheter tip culture and blood culture were both negative. No local phlebitis occurred in the affected infant. The *Guide for prevention and control of catheter-related bloodstream infection 2011 version*^[19]^ recommends that PICC should never be withdrawn simply due to fever and comprehensive evaluation should be made based on the clinical manifestations on whether or the withdrawal is necessary (type II). When patients suffer from phlebitis (erythema, swelling, pain, or palpable venous cord), infections or catheter dysfunctions, PICC should be withdrawn in time (type IB). During the treatment for very-low/ultra-low birth weight infants, unnecessary catheter withdrawal increases the possibility of re-insertion and unnecessary pain to them. Therefore, the decision of whether a catheter should be withdrawn should be made after careful and comprehensive evaluations.

In our study, 1 case had arrhythmia due to incorrect PICC insertion into the heart chamber. The patient was a low birth weight infant and underwent PICC insertion 2^+^ days after the birth. Chest X-ray after the initial insertion indicated that the PICC line was located at the upper edge of the 5th cervical vertebral body and had been incorrectly placed into the jugular vein; therefore, the PICC was withdrawn by 0.5 cm and routine care was given afterwards. On day 4 after the insertion, the infant suddenly suffered from shortness of breath and significant difficulty breathing while their heart rate slowed to 60 to 70 beats/min and the electrocardiogram (ECG) showed frequent atrial premature contractions (bigeminy), junctional escape beats, Q-T prolongation, flat and low T wave, and sinus bradycardia. Chest X-rays showed that the PICC line was located at the right transverse process of the 6th thoracic vertebra. Thus, PICC migration and incorrect insertion into the heart chamber were considered. The infant's symptoms were improved after immediate catheter withdrawal and nutritional myocardial therapy. Reasons for the incorrect insertion of the catheter tip into the jugular vein but not the central vein during the initial PICC insertion were possibly due to the incorrect position of the infant during the insertion. On the same day, the PICC was withdrawn by 0.5 cm and re-fixated externally and routine care was given afterwards. However, the catheter tip migrated into the heart chamber later. Difference in locations of the catheter tip in infants may result from the inadequate catheter fixation at the body surface or voluntary limb movements and passive position changes of the newborns. It was reported that the position of the PICC tip may move when the arm moves from abduction to adduction; while the difference in movement is usually within 2.0 cm.^[20]^ However, even a change of 0.5 to 1 cm will be great for a newborn, especially a very-low birth weight infant, since the position of the catheter tip can be very different. For newborns, especially a very-low birth weight infant, accurate positioning of the PICC tip is extremely important. If a severe arrhythmia appears after catheter insertion, professionals should be immediately aware that it may be due to an excessively deep catheter position and the catheter should be withdrawn immediately. Thus, continuous ECG monitoring is required after the catheter insertion, and X-rays should be taken immediately to identify the position of the catheter tip.

This study has some limitations. As a retrospective study the analysis is limited, and we were unable to undertake multivariate analysis to find risk factors for complications. The sample size for this technique at our center is relatively small; we will further improve the data recording in the future applications so as to accumulate more clinical experiences.

In conclusion, PICC offers a circulating pathway for the successful rescue and treatment of newborns, especially the very-low birth weight and critically ill infants, while avoiding issues such as infections and pain caused by repeated punctures. However, nursing measures of the maintenance of body temperature and the evaluation of blood vessels were important conditions for improving the success rate of one puncture in critically ill neonates. PICC catheterization as early as 48 hours will not increase the difficulty of PICC puncture. Nor did it increase the incidence of PICC complications.

## Author contributions

**Conceptualization:** Xia Cao, Tian Shi.

**Data curation:** Renfeng Li, Xia Cao.

**Formal analysis:** Tian Shi.

**Writing – original draft:** Renfeng Li, Lei Xiong.

**Writing – review & editing:** Lei Xiong.
